# Anxiety and Depression Associated With the Dependent Use of Generative AI in Medical Students: Cross-Sectional Study

**DOI:** 10.2196/82667

**Published:** 2026-05-05

**Authors:** Janett V Chavez Sosa, Salomon Huancahuire-Vega

**Affiliations:** 1 Unidad de Salud, Escuela de Posgrado, Universidad Peruana Unión (UPeU), Lima, Peru; 2 Basic Sciences Department, Escuela de Medicina Humana, Facultad de Ciencias de la Salud, Universidad Peruana Unión (UPeU), Carretera central Km 19, Ñaña, Lima, Peru, 51 997574011

**Keywords:** artificial intelligence, AI, mental health, anxiety disorders, depressive disorders, medical students

## Abstract

**Background:**

The growing integration of artificial intelligence (AI) in higher education has transformed learning processes but also raised concerns about potential mental health risks. Medical students represent a particularly vulnerable group due to high academic stress and increasing reliance on generative AI tools for study and decision-making tasks. Despite this, the relationship between AI dependence and psychological distress remains underexplored in Latin American contexts.

**Objective:**

This study aimed to evaluate the association between generative AI dependence and levels of stress, anxiety, and depression among medical students.

**Methods:**

A cross-sectional study was conducted with 187 human medicine students from a Peruvian university during the first academic semester of 2025. The Dependence on Artificial Intelligence Scale and the Depression, Anxiety, and Stress Scale–21 were applied. Negative binomial regression models, both crude and adjusted for sex, age, income, and year of study, were used to assess associations, reporting rate ratios (RRs) and 95% CIs.

**Results:**

Participants had a median age of 22 (IQR 19‐24) years, and 58.8% (110/187) were female. The median Dependence on Artificial Intelligence Scale score was 10 (IQR 7‐14). Generative AI dependence showed significant correlations with anxiety (ρ=0.336, 95% CI 0.22‐0.44) and depression (ρ=0.316, 95% CI 0.20‐0.43) and a smaller correlation with stress (ρ=0.277, 95% CI 0.16‐0.39). In the adjusted regression models, each 1-point increase in generative AI dependence was associated with a 5% higher expected anxiety score (RR 1.05, 95% CI 1.01‐1.09; *P*=.01) and a 4% higher depression score (RR 1.04, 95% CI 1.01‐1.08; *P*=.03), whereas the association with stress was positive but nonsignificant (RR 1.03, 95% CI 1.00‐1.07; *P*=.08). Fifth-year students had significantly greater anxiety levels than their sixth-year peers (RR 1.82, 95% CI 1.09‐3.01; *P*=.02). No significant effects were observed for sex, age, or income.

**Conclusions:**

This study empirically examined generative AI dependence as a distinct behavioral construct and its association with mental health symptoms in medical students. Unlike prior research, this study evaluated psychological dependence on generative AI and modeled its relationship with anxiety and depression using appropriate count-based regression techniques. By providing early evidence from a Latin American context, it contributes to the emerging field of digital mental health and medical education research. These findings underscore the need for universities to promote balanced and responsible AI use, integrate digital literacy with mental health support strategies, and develop preventive policies that mitigate potential maladaptive reliance on generative AI tools.

## Introduction

In recent years, artificial intelligence (AI) has been incorporated into various fields, including mental health by offering innovative tools for the detection and prevention of and intervention in psychological disorders. Applications such as therapeutic chatbots, natural language analysis, and machine learning algorithms have demonstrated utility in identifying emotional states, affective regulation, and monitoring mental well-being in real time [1,2]. However, the intensive and daily use of AI, especially in academic and technological environments, has generated new problems, including psychological dependence, reduced self-efficacy, and mental health deterioration among young university students [3,4].

AI is defined as a set of computational systems capable of performing tasks that traditionally require human intelligence, such as reasoning, problem-solving, and autonomous learning. Within this broad field, generative AI represents a recent advancement that uses language models and deep learning techniques to create new content in the form of text, images, code, or sound based on human instructions (prompts). Its most widespread form in the educational context consists of AI-powered chatbots such as ChatGPT, Copilot, or Gemini, which enable conversational interaction and can provide explanations, draft texts, synthesize information, or solve academic exercises [5]. Although these technologies have positively transformed learning, writing, and programming processes, concerns have also been raised about their potential negative effects, including the loss of cognitive skills, reduced critical thinking, and the development of excessive dependence on such tools [6].

In this context, dependence on generative AI is conceived as a compulsive tendency or psychological need to rely on automated systems to perform tasks, make decisions, or validate one’s own performance. This phenomenon shares features with behavioral addictions described in the *Diagnostic and Statistical Manual of Mental Disorders, Fifth Edition*, such as tolerance, the search for external validation, and anxiety associated with technological disconnection. The Dependence on Artificial Intelligence Scale (DIA) used in this study is grounded in these conceptual criteria, encompassing dimensions such as perceived vulnerability, performance-related concern, the need to maintain an updated personal image, the search for external validation, and fear of personal obsolescence [7]. AI dependence represents a qualitatively distinct form of psychological reliance characterized by interactive, generative, and cognitively assistive dynamics. Unlike general technology dependence, generative AI dependence emerges from dynamic human-machine interactions that engage both cognitive and emotional processes through adaptive, generative feedback [8].

From a theoretical perspective, generative AI dependence integrates cognitive, emotional, and behavioral mechanisms. According to cognitive offloading theory, delegating reasoning and decision-making to generative AI may undermine self-efficacy and increase stress when independent thinking is required. Behavioral addiction theory explains how AI’s rewarding and feedback-rich interactions can promote compulsive use, whereas human-computer interaction research highlights the illusion of cognitive companionship that reinforces overreliance and emotional validation seeking [9]. Together, these frameworks suggest that AI dependence is a distinct form of psychological reliance linked to stress, anxiety, and depression through reduced autonomy and maladaptive coping.

On the other hand, the current literature recognizes that global mental health is in crisis. It is estimated that mental disorders represent 32% of the years lived with disability worldwide, with a high prevalence in people under 25 years of age [10]. This problem is exacerbated in academically demanding populations, such as medical students, who face high levels of stress, anxiety, and depression due to academic pressure, information overload, and institutional competitiveness [11,12]. In this context, generative AI has been incorporated both as an educational tool and as a tool for emotional self-regulation; however, its constant use could generate patterns of dependence that, far from improving well-being, could contribute to greater emotional dysregulation and isolation [13,14].

Recent studies have shown that excessive use of generative AI–based digital technologies can be associated with negative mental health consequences, such as cognitive fatigue, difficulty disconnecting, decreased sleep quality, and lower frustration tolerance [15,16]. In addition, the literature shows ethical concerns about privacy, user autonomy, and the possible replacement of human contact with interactions with automated systems [10,11]. In particular, vulnerable subgroups such as students with a history of mental disorders may experience a greater negative impact derived from the excessive use of generative AI in academic or personal contexts [15].

Despite its transformative potential, the implementation of generative AI technologies in mental health has not been fully understood or regulated [17]. There are still significant knowledge gaps around its overuse, especially among young adults immersed in digitized university environments [18]. The literature shows that, although there are obvious benefits, latent risks are also recognized, including psychological dependence and the decrease in critical thinking in the face of the constant use of predictive tools and automated decision-making [19,20].

On the other hand, the literature recognizes that future health care professionals are a vulnerable population experiencing anxiety or depression and that factors such as academic overload, professional uncertainty, and the COVID-19 pandemic have exacerbated these disorders [17]. Studies among Peruvian medical students have shown that excessive use of generative AI can erode motivation and essential cognitive skills [21]. Consistently, editorials on medical education warn that blind reliance on automated systems can reduce critical thinking and decision-making capacity, making it urgent to establish regulatory frameworks and promote the ethical integration of AI into the curriculum [22,23].

Given this background and the absence of empirical studies linking generative AI dependence with symptoms of anxiety and depression in medical students, the following hypothesis was formulated: greater dependence on generative AI will be positively associated with higher levels of stress, anxiety, and depression in medical students.

## Methods

### Design, Population, and Sample

This study had an observational and cross-sectional design. The population consisted of 747 human medicine students enrolled in a private university in Lima during the first academic semester of 2025. A nonprobabilistic quota sampling was used, distributed proportionally according to the year of study (from first to sixth year) to ensure an equitable representation of the different training stages. The seventh year (medical internship) was excluded due to the care burden and the limited availability of time for students at that stage.

The inclusion criteria were adults over 18 years of age enrolled and with a full academic load in the current semester and agreement to participate voluntarily by signing the informed consent form. Exclusion criteria were presenting a self-declared clinical diagnosis of a mental disorder (such as major depression, anxiety disorders, and bipolar disorder, among others), being under pharmacological treatment with psychotropics or antidepressants, not signing the informed consent form, and being a seventh-year student.

**Figure 1.**
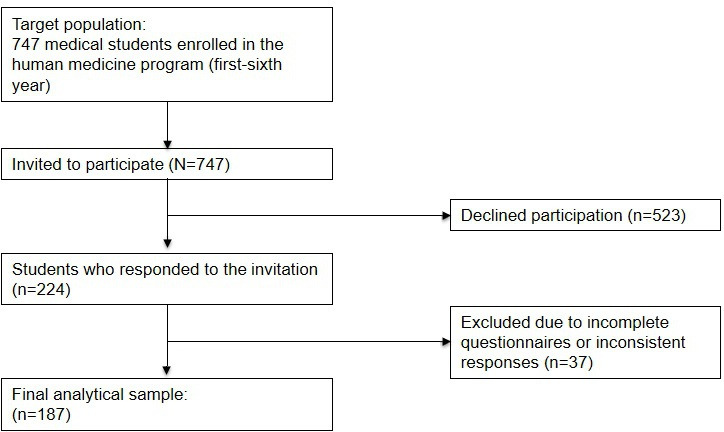
Flow diagram of participants included in the study.

On the basis of the quota sampling, a sample of 224 students was projected, distributed proportionally according to the number of students enrolled per academic year. However, effective data collection was determined by participants’ voluntary acceptance and availability, resulting in a final sample of 187 students distributed as follows: 28 (15%) first-year students, 42 (22.5%) second-year students, 22 (11.8%) third-year students, 14 (7.5%) fourth-year students, 32 (17.1%) fifth-year students, and 49 (26.2%) sixth-year students (Figure 1). This study followed the recommendations of the STROBE (Strengthening the Reporting of Observational Studies in Epidemiology) guidelines to ensure adequate and complete reporting of cross-sectional studies.

Although the sample size achieved was smaller than initially projected, a post hoc estimate of statistical power was made in negative binomial regression models. From the observed coefficients (*B*=0.0-0.05) and low SEs (approximately 0.018 to 0.020), it was estimated that the statistical power was greater than 80%, a value considered adequate to detect real effects with an acceptable probability of type 2 error (β<.20). This supports the robustness of the findings and justifies the inferential validity of the study even with a small sample.

### Instruments

To evaluate the variable of dependence on AI, the DIA was used, developed by Morales-García et al [21] in 2024 in Peru. This instrument was designed for the university population, is unidimensional, and is composed of 5 items. Responses are provided on a 5-point Likert-type scale ranging from 1 (“Totally false to me”) to 5 (“Describes me perfectly”). The total score is obtained through the direct summation of the 5 items, with a possible range of 5 to 25 points. The higher the score, the higher the level of psychological dependence on AI. This instrument has demonstrated good construct validity (comparative fit index=0.99; Tucker-Lewis index=0.98; root mean squared error of approximation=0.05) and reliability (Cronbach α=0.87; ω=0.87).

To measure mental health, the Depression, Anxiety, and Stress Scale–21 (DASS-21) was used, developed by Lovibond and Lovibond [24] in 1995 in Australia and internationally validated in the university population [25]. The instrument consists of 21 items divided into 3 dimensions: stress (7 items: 1, 6, 8, 11, 12, 14, and 18), anxiety (7 items: 2, 4, 7, 9, 15, 19, and 20), and depression (7 items: 3, 5, 10, 13, 16, 17, and 21). Each item is rated on a 4-point Likert-type scale from 0 (“Nothing has happened to me”) to 3 (“A lot has happened to me” or “Almost always”) depending on the symptoms experienced in the previous week. The final score on each dimension is obtained by adding the corresponding items and multiplying by 2, obtaining a range between 0 and 42 points per subscale. The higher the score, the greater the severity of the symptom in each category (stress, anxiety, or depression). This scale presents high levels of reliability in the university population, with Cronbach α coefficients greater than 0.85 for each subscale in various international and Latin American studies.

The internal consistency of both instruments was assessed using the Cronbach α coefficient in this study sample. The DIA showed good reliability (Cronbach α=0.859). For the DASS-21, overall internal consistency was excellent (Cronbach α=0.953). By subscale, a Cronbach α value of 0.901 was obtained for stress, a Cronbach α value of 0.855 was obtained for anxiety, and a Cronbach α value of 0.887 was obtained for depression. These results confirm the adequate internal consistency of both instruments in the sample of Peruvian medical students.

Additionally, sociodemographic information was collected, including sex, age, place of birth, marital status, employment status, family income, year of study, current place of residence, internet connectivity, and reasons for the use of AI.

The “reasons for the use of AI” item was designed to explore students’ main motivations for interacting with generative AI tools in academic and personal contexts. Response categories were based on previous literature on motives for technology use and on pilot observations with Peruvian university students. Drawing on self-determination theory [26], which distinguishes between intrinsic and extrinsic motivation, and the uses and gratifications framework, which focuses on the functional ends of media use, motives were grouped into three domains: (1) academic and learning support (summarizing content, resolving doubts, and writing papers), (2) productivity and efficiency (writing assistance, idea generation, and optimizing study time), and (3) personal or recreational use (curiosity, entertainment, and general exploration) [27].

### Procedure

Data collection was carried out between March 2025 and June 2025 using an online self-administered questionnaire through Google Forms. This questionnaire included an initial section of project information and informed consent. The form link was disseminated through institutional means and authorized academic channels. Data were analyzed immediately after collection, and manuscript preparation occurred later in 2025.

### Statistical Analysis Plan

The statistical analysis was carried out using the SPSS Statistics software (version 29; IBM Corp). First, the database was debugged to identify and correct lost or inconsistent values or outliers. The assumption of normality for all continuous variables was assessed using the Shapiro-Wilk test, as well as through skewness and kurtosis measurements. The results indicated nonnormal distributions across all scales (*P*<.001 in the Shapiro-Wilk test). Specifically, the skewness and kurtosis values were 0.69 and −0.19 for AI dependence, 0.72 and 0.05 for the total DASS-21 score, 0.88 and 0.31 for anxiety, 1.02 and 0.50 for depression, and 0.44 and −0.47 for stress, respectively (). Visual inspection of the histograms also revealed positive skewness in the scores, confirming the lack of normality. Therefore, nonparametric statistics were chosen for the bivariate analyses (Mann-Whitney *U* and Kruskal-Wallis tests), and the results were reported as medians and IQRs. Absolute and relative frequencies were used to describe the categorical variables.

In the bivariate analysis phase, the association between generative AI dependence and mental health dimensions (stress, anxiety, and depression) was examined applying the Spearman ρ correlation test due to the nonparametric nature of the data. Similarly, the Mann-Whitney *U* and Kruskal-Wallis tests were used to compare the scores of the dependent variables according to the nature of the covariates.

As the outcome variables (stress, anxiety, and depression) were treated as counting variables and presented overdispersion, it was decided to apply negative binomial regression models in the multivariate analysis. For each dependent variable, 2 models were estimated: a crude model that included only the independent variable (dependence on generative AI) and an adjusted model that considered potentially confounding variables (sex, age, and year of study). Rate ratios (RRs), 95% CIs, and *P* values were reported considering a level of statistical significance of a *P* value of less than .05. Given the cross-sectional design, exponentiated coefficients from the negative binomial models represent ratios of expected mean symptom scores rather than incidence RRs over time.

Finally, the global fit of the models was evaluated by comparing the log-likelihood between raw and adjusted models. An improvement of the fit in the adjusted models was obtained, evidenced by a decrease in the value of the log-likelihood (−2LL). Similarly, the Nagelkerke pseudo-*R*^2^ was used, whose values were 0.042 for the stress model, 0.067 for the anxiety model, and 0.059 for the depression model, indicating a low but acceptable explanatory capacity of the model on the variability of the psychological symptoms evaluated.

### Ethical Considerations

This study was reviewed and approved by the Research Ethics Committee of Universidad Peruana Unión (REC; resolution number 2025-CEUPeU-030). All procedures were conducted in accordance with the ethical standards of the responsible institutional committee and with the principles of the Declaration of Helsinki (2025-CEUPeU-030). Participation was voluntary, and all participants provided electronic informed consent prior to enrollment after receiving detailed information about the study’s objectives, procedures, potential risks, and benefits. No financial or other compensation was provided to participants. To ensure privacy and confidentiality, data were collected anonymously through a secure online platform, and no identifying information was recorded. All data were stored in password-protected files accessible only to the research team. No identifiable images or personal information are included in the manuscript or supplementary materials.

## Results

A total of 187 medical students participated in the study. Most (n=110, 58.8%) were female, and the median age was 22 (IQR 19‐24) years. Regarding their place of origin, 47.6% (n=89) came from the interior of the country, 42.2% (n=79) came from the Lima Metropolitan Area, and 10.2% (n=19) came from abroad. Most reported not having a partner (n=146, 78.1%) and not having a job (n=174, 93%). Regarding family income, 75.9% (n=142) reported an income greater than the country’s minimum wage (approximately US $315). The study years with the highest representation were the sixth (n=49, 26.2%), second (n=42, 22.5%), and fifth (n=32, 17.1%) years. Regarding current residence, 54% (n=101) lived in rented accommodations, 32.6% (n=61) lived with relatives, and 13.4% (n=25) lived in a university residence. A total of 79.7% (n=149) reported having a stable internet connection. Regarding the reasons for the use of AI, academic tasks (n=91, 48.7%) and personal learning (n=81, 43.3%) predominated (Table 1).

**Table 1.** General characteristics of medical students (N=187).

Variables	Value
Sex, n (%)	
Female	110 (58.8)
Male	77 (41.2)
Age (y), median (IQR)	22 (19-24)
Place of origin, n (%)	
Lima	79 (42.2)
Interior of the country	89 (47.6)
Abroad	19 (10.2)
Marital status, n (%)	
With a partner	41 (21.9)
Without a partner	146 (78.1)
Employment status, n (%)	
Working	13 (7)
Not working	174 (93)
Family income, n (%)	
Over the minimum wage	142 (75.9)
Below the minimum wage	45 (24.1)
Year of study, n (%)	
First	28 (15)
Second	42 (22.5)
Third	22 (11.8)
Fourth	14 (7.5)
Fifth	32 (17.1)
Sixth	49 (26.2)
Current residence, n (%)	
Rental	101 (54)
University residence	25 (13.4)
With family	61 (32.6)
Internet connection, n (%)	
Yes, stable	149 (79.7)
Yes but unstable	38 (20.3)
Reasons for using AI^a^, n (%)	
Academic assignments	91 (48.7)
Personal learning	81 (43.3)
Entertainment	4 (2.1)
Health consultations	2 (1.1)
Other	9 (4.8)

a AI: artificial intelligence.

Regarding the main variables of the study, the median dependence on generative AI score was 10 (IQR 7‐14) points, with a range of 5 to 25 points. The median total score on mental health (DASS-21) was 15 (IQR 6‐25) points, with stress being the dimension with the highest score (median 6, IQR 2-11), followed by anxiety (median 4, IQR 1-7) and depression (median 4, IQR 2-8; Table 2).

**Table 2.** Descriptive statistics of the main variables.

Variables	Values, median (IQR; 95% CI)	Values, range
Generative AI^a^ dependence score (5-25)	10 (7-14; 9-11)	5‐25
Mental health score (0-63; DASS-21^b^)	15 (6-25; 13-17)	0‐61
Stress (0-42)	6 (2-11; 6-7)	0‐21
Anxiety (0-42)	4 (1-7; 3-5)	0‐19
Depression (0-42)	4 (2-8; 3-5)	0‐21

a AI: artificial intelligence.

b DASS-21: Depression, Anxiety, and Stress Scale–21.

In the bivariate analysis, significant differences were found in stress levels according to sex, being higher in women (*P*=.03). Differences were also observed in anxiety (*P*=.01) and depression (*P*=.04) according to income, being greater in those who reported an income below the minimum wage. Similarly, the year of study was significantly associated with stress (*P*=.01) and anxiety (*P*<.001), with higher scores being observed in second- and fifth-year students. No significant associations were observed with other variables, such as age, marital status, employment status, or type of residence (Table 3).

**Table 3.** Bivariate analysis according to mental health (Depression, Anxiety, and Stress Scale–21; DASS-21) in medical students.

Variables	Mental health (DASS-21)					
	Stress (0-42), median (IQR)	*P* value	Anxiety (0-42), median (IQR)	*P* value	Depression (0-42), median (IQR)	*P* value
Sex		.03		.19		.69
Female	7 (3-12)		5 (1-8)		4 (2-9)	
Male	6 (2-9)		4 (2-7)		3 (1-8)	
Age (y)	0.008^a^	.91	–0.054^a^	.46	–0.037^a^	.61
Place of origin		.20		.23		.47
Lima	7 (2.5‐11)		5 (1-9)		4 (2-8)	
Interior of the country	6 (3‐10.5)		4 (1-7)		4 (2‐7.5)	
Abroad	4 (0‐8.5)		3 (0‐6)		3 (0‐7)	
Marital status		.88		.37		.52
With a partner	6 (2-10)		4 (2-7)		3 (1-7)	
Without a partner	7 (3-12)		5 (1-8)		4 (2-9)	
Employment status		.57		.59		.76
Working	7 (3-12)		5 (2-8)		4 (2-9)	
Not working	6 (2-10)		4 (1-7)		3 (1-8)	
Family income		.09		.01		.04
Over the minimum wage	6 (2-10)		4 (1-7)		4 (1-8)	
Below the minimum wage	8 (3-12)		7 (1-8)		5 (1-8)	
Year of study		.01		<.001		.06
First	4 (1‐6.5)		3.5 (1-5)		3 (1‐6.5)	
Second	7.5 (4-12)		5.5 (2-9)		5 (2-9)	
Third	5 (1-11)		2.5 (0‐7)		3 (0‐8)	
Fourth	7 (6-11)		5.5 (2-7)		5 (2-8)	
Fifth	8.5 (6-12)		6.5 (4-9)		6 (3-11)	
Sixth	6 (2-10)		3 (0‐5)		3 (1-5)	
Current residence		.97		.81		.92
Rental	6 (2-11)		4 (1-7)		4 (1.0‐8.0)	
University residence	6 (4-11)		4 (1‐6.5)		4 (2‐8.5)	
With family	7 (3-11)		4 (2-9)		4 (2-7)	
Internet connection		.89		.57		.94
Yes, stable	6 (2-11)		4 (1‐7.5)		4 (1.5‐8)	
Yes but unstable	6 (3.5‐10)		5 (2-7)		4 (2‐7.25)	
Reasons for using AI^b^		.42		.08		.13
Academic assignments	6 (2‐10.5)		4 (1-7)		4 (2-7)	
Personal learning	6 (3-11)		5 (1-7)		4 (2-8)	
Entertainment	9 (7.5‐11.5)		10.5 (8.5‐13)		9 (7-11)	
Health consultations	7 (0‐14)		5.5 (0‐11)		10.5 (3-18)	
Other	1 (0‐8)		1 (1-3)		2 (1-2)	

a Spearman ρ.

b AI: artificial intelligence.

A positive and statistically significant correlation was found between AI dependence and all 3 mental health outcomes. Specifically, AI dependence showed a small correlation with stress (ρ=0.277; *P*<.001) and moderate correlations with anxiety (ρ=0.336; *P*<.001) and depression (ρ=0.316; *P*<.001). These findings indicate that higher levels of dependence on generative AI are associated with greater psychological distress, particularly anxiety and depression symptoms. In contrast, age was not significantly correlated with any of the DASS-21 outcomes. The correlations between age and stress (ρ=–0.118; *P*=.09), anxiety (ρ=–0.102; *P*=.13), and depression (ρ=–0.087; *P*=.21) were negative but of very small magnitude and statistically nonsignificant (Table 4).

**Table 4.** Spearman correlations among generative artificial intelligence (AI) dependence, age, and mental health outcomes (Depression, Anxiety, and Stress Scale–21)^a^.

Variable	Spearman ρ (95% CI)	*P* value	Effect size interpretation
AI dependence × stress	0.277 (0.16 to 0.39)	<.001	Small
AI dependence × anxiety	0.336 (0.22 to 0.44)	<.001	Moderate
AI dependence × depression	0.316 (0.20 to 0.43)	<.001	Moderate
Age × stress	–0.118 (–0.25 to 0.02)	.09	Very small
Age × anxiety	–0.102 (–0.23 to 0.03)	.13	Very small
Age × depression	–0.087 (–0.21 to 0.05)	.21	Very small

a Interpretation according to Cohen [28] : a |ρ| value of 0.10 corresponds to a small effect size, 0.30 corresponds to a moderate effect size, and 0.50 corresponds to a large effect size.

In the multivariate analysis, in the adjusted model, it was observed that, for each additional point in the AI dependence score, the expected anxiety score increased by 5% (RR 1.05, 95% CI 1.01‐1.09; *P*=.01) and the depression score increased by 4% (RR 1.04, 95% CI 1.01‐1.08; *P*=.03). For stress, the relationship was positive but not significant (RR 1.03, 95% CI 1.00‐1.07; *P*=.08). Furthermore, fifth-year students had an 82% higher expected anxiety score than sixth-year students (RR 1.82, 95% CI 1.09‐3.01; *P*=.02). No significant associations were found between sociodemographic variables (sex, age, and monthly income) and levels of stress, anxiety, or depression (Table 5). These results suggest that dependence on AI may be a relevant risk factor for mental health, especially in the components of anxiety and depression, in medical students.

**Table 5.** Multivariate analysis according to mental health (Depression, Anxiety, and Stress Scale–21) in medical students.

Variable	Stress		Anxiety		Depression	
	*B*^a^ (95% CI)	RR^b^ (95% CI)	*B* (95% CI)	RR (95% CI)	*B* (95% CI)	RR (95% CI)
AI^c^ dependence	0.03 (–0.00 to 0.07)	1.03 (1.00 to 1.07)	0.05 (0.01 to 0.09)	1.05 (1.01 to 1.09)	0.04 (0.01 to 0.08)	1.04 (1.01 to 1.08)
Sex (reference=male)	0.24 (–0.08 to 0.57)	1.27 (0.92 to 1.77)	0.15 (–0.19 to 0.48)	1.16 (0.83 to 1.61)	0.01 (–0.33 to 0.34)	1.01 (0.72 to 1.40)
Age	–0.04 (–0.10 to 0.01)	0.96 (0.91 to 1.01)	–0.01 (–0.07 to 0.04)	0.99 (0.93 to 1.04)	–0.04 (–0.10 to 0.02)	0.96 (0.91 to 1.02)
Income (reference=over the minimum wage)	0.16 (–0.21 to 0.54)	1.17 (0.81 to 1.72)	0.30 (–0.08 to 0.69)	1.35 (0.92 to 1.99)	0.30 (–0.09 to 0.69)	1.35 (0.91 to 1.99)
Year of study (reference=sixth)						
First	–0.54 (–1.14 to 0.07)	0.58 (0.32 to 1.07)	0.06 (–0.57 to 0.68)	1.06 (0.56 to 1.97)	–0.07 (–0.69 to 0.56)	0.93 (0.50 to 1.75)
Second	–0.10 (–0.64 to 0.45)	0.91 (0.53 to 1.57)	0.34 (–0.22 to 0.90)	1.40 (0.80 to 2.46)	0.00 (–0.58 to 0.58)	1.00 (0.56 to 1.79)
Third	–0.23 (–0.82 to 0.36)	0.79 (0.44 to 1.43)	0.08 (–0.53 to 0.70)	1.08 (0.59 to 2.01)	–0.07 (–0.68 to 0.54)	0.93 (0.51 to 1.71)
Fourth	0.01 (–0.66 to 0.67)	1.01 (0.52 to 1.95)	0.40 (–0.28 to 1.09)	1.49 (0.75 to 2.98)	0.26 (–0.43 to 0.93)	1.30 (0.65 to 2.53)
Fifth	0.17 (–0.32 to 0.65)	1.19 (0.72 to 1.91)	0.60 (0.09 to 1.10)	1.82 (1.09 to 3.01)	0.45 (–0.05 to 0.95)	1.57 (0.95 to 2.58)

a Regression coefficient (log rate).

b RR: rate ratio.

c AI: artificial intelligence.

## Discussion

### Principal Findings

This study found that dependence on generative AI was significantly associated with higher levels of anxiety and depression in medical students even after adjusting for sociodemographic variables such as sex, age, income, and year of study. The effect sizes, while statistically significant, were of small to moderate magnitude, with RRs ranging from 1.03 to 1.05, suggesting that, for each 1-point increase in AI dependence, there was a 3% to 5% increase in the expected levels of anxiety and depression. This finding highlights that, although the associations are modest, they are consistent and meaningful in the context of behavioral and psychological research. These findings are meaningful when interpreted through the theoretical framework proposed. The findings support the notion that generative AI dependence represents a distinct form of digital reliance characterized by interactive, generative, and cognitively assistive mechanisms rather than passive technology use, especially in highly demanding academic contexts and when their use becomes compulsive or replaces meaningful human interactions [29,30].

### Comparison to Prior Work and Possible Explanation of Associations

These results are consistent with those of the study by Huang et al [16], who, in a longitudinal model, found that mental health problems such as anxiety and depression predicted a subsequent increase in AI dependence mainly when students used these tools for purposes of escape or social interaction. Although AI may seem like an accessible solution, it could also reinforce patterns of use that aggravate psychological distress if not adequately regulated. Along the same lines, another study reported that nursing students with higher levels of anxiety and depression showed more positive attitudes toward the use of AI-based mental health tools [17]. While this indicates an openness toward digital solutions, it could also reflect a functional dependence or a search for support in less stigmatizing environments than traditional psychological care. This trend could become a gateway to technological dependence if not accompanied by critical digital literacy and career guidance. Small effect sizes, as observed in this study, are common in psychological and educational studies examining multifactorial constructs such as mental health and technology use. Importantly, even small effects may have practical significance when considered across large populations of students exposed to AI-based technologies.

On the other hand, it has been evidenced that psychological distress predicts the most frequent use of digital mental health applications, associated with factors such as digital skills, attitudes toward mental health, and perception of the usefulness of AI [31,32]. Complementarily, a bibliometric analysis showed that generative AI has emerged as a promising tool to support the mental health of university students, especially in the detection of anxiety and depression through big data, machine learning, and portable technologies [33,34]. However, the aforementioned study also warns that, despite the advantages in terms of accessibility and diagnostic accuracy, there are still methodological, ethical, and practical challenges that require constant regulation and evaluation. This bibliometric perspective supports the urgency of designing educational strategies that prepare students for the responsible, balanced, and conscious use of generative AI, especially in academic contexts, through psychoeducational interventions that address both students’ emotional well-being and their digital skills [35].

Some mechanisms could explain the association between generative AI dependence and anxiety. One of them is digital addiction, which can generate a constant need for immediate feedback and gratification, exacerbating worry, irritability, and the feeling of loss of control [36]. The observed associations align with cognitive offloading theory, suggesting that the habitual delegation of intellectual tasks to AI systems may diminish users’ confidence in their own cognitive abilities. Over time, this can induce performance anxiety and feelings of incompetence when independent reasoning is required. Simultaneously, the behavioral addiction model helps explain how AI’s immediate feedback and personalized reinforcement can activate reward pathways, promoting compulsive use similar to other nonsubstance addictions [37].

Regarding depression, it has been suggested that compulsive use of AI can foster social isolation and the replacement of human contact with automated interactions, which weakens affective bonds and decreases perceived emotional support [31,37]. From the human-computer interaction perspective, the interactive and adaptive nature of generative AI, capable of conversational empathy and tailored assistance, creates a pseudosocial dynamic that may satisfy emotional needs for support or validation. This emotional reinforcement loop may partially account for the observed link between generative AI dependence and depressive symptoms as users increasingly turn to AI for reassurance rather than to human connection or self-reflection. These factors, added to the typical academic load of the medical career, can facilitate the appearance of depressive symptoms.

While this cross-sectional study cannot establish causal direction, the relationship between generative AI dependence and mental health is likely bidirectional. It is plausible that students with preexisting anxiety or depressive symptoms may turn to generative AI as a coping mechanism, seeking reassurance, cognitive assistance, or emotional relief through its adaptive responses. However, over time, such reliance may contribute to a reinforcing feedback loop: academic pressure and emotional distress increase generative AI use for cognitive offloading, which gradually erodes self-efficacy, autonomy, and critical thinking; this decline, in turn, exacerbates psychological distress and further deepens dependence on AI tools. This model aligns with theoretical frameworks of behavioral reinforcement and cognitive offloading, suggesting that generative AI dependence may function simultaneously as a symptom and a stress amplifier. Understanding these reciprocal pathways is critical to advancing digital mental health research.

Students recognize the benefits of AI in medical practice; however, they also express concerns about its possible negative association with the physician-patient relationship, confidentiality, and job security, among other aspects [38]. These ambivalences could increase stress among future physicians as they face a professional environment in constant technological transformation for which they often do not feel fully prepared. In our study, the nonsignificant association between AI dependence and stress suggested a complex relationship in which generative AI may temporarily reduce academic stress by improving efficiency yet simultaneously intensify deeper emotional vulnerabilities such as anxiety and self-doubt. This pattern reflects an avoidance-based coping mechanism, using generative AI to manage pressure while reinforcing dependence and emotional dysregulation over time, warranting further longitudinal investigation [39].

In addition to the primary variable, it was identified that fifth-year students reported higher levels of anxiety than those in the sixth year, which can be interpreted from a transitional perspective. Almusharraf et al [40] found that perceptions of AI among health sciences students vary by academic year and level of exposure to these technologies. The higher probability of anxiety observed among fifth-year students may reflect the unique transitional stressors inherent to this stage of Peruvian medical education. The fifth year typically marks the shift from academic coursework to intensive clinical clerkships, during which students assume greater patient care responsibilities under supervision and are continuously evaluated on clinical competence. This period also coincides with preparation for the National Medical Examination, a high-stakes assessment that determines eligibility for the mandatory internship (sixth year) [41]. The simultaneous exposure to clinical uncertainty, extended working hours, and academic evaluation pressure can amplify anxiety symptoms as students face the dual challenge of consolidating theoretical knowledge while adapting to real-world medical practice. Similar patterns have been reported in other settings where transitions to clinical training and licensing examinations coincide [42]. This finding suggests that there are critical periods within medical training that require specific preventive interventions in mental health.

### Implications

The explicit interpretation of effect sizes in this study provides a more precise understanding of the strength and practical significance of the associations between AI dependence and mental health outcomes. Although the observed associations were of small to moderate magnitude, they point toward a broader maladaptive behavioral pattern associated with excessive reliance on generative AI for cognitive and emotional regulation. This pattern mirrors those identified in digital dependence literature, where overuse fosters avoidance behaviors, reduced self-efficacy, and emotional dysregulation, factors that contribute to heightened anxiety and depression. Framing these findings within this broader context situates this study within the emerging field of digital mental health, emphasizing that the implications of AI dependence extend beyond individual distress to reflect changing patterns of learning, coping, and self-perception in technologically mediated environments. From a public health and educational perspective, even small increases in distress linked to AI overuse can have meaningful cumulative consequences for academic functioning and well-being. Universities must implement comprehensive student welfare policies that include psychological counseling, active listening spaces, and psychosocial risk monitoring, especially in careers with high emotional demand such as medicine.

Future research should explore these associations through longitudinal and mixed methods designs, incorporating objective behavioral indicators and cross-cultural analyses to better understand how AI dependence interacts with psychological distress over time. Such approaches would inform evidence-based frameworks for promoting healthy digital engagement and mitigating the risks of maladaptive AI use in higher education.

### Limitations

This study has some limitations. As it had a cross-sectional design, it did not allow for establishing causal relationships between generative AI dependence and mental health indicators. Additionally, the measurement was based on self-reports, which could introduce social desirability biases or lead to the underestimation of psychological distress. To mitigate the social desirability bias, future research should address these limitations by triangulating subjective reports with objective indicators such as physiological or behavioral data, applying anonymous digital survey methods to reduce social pressure, and including validity or social desirability scales to statistically control for response bias. Dependence on generative AI for tasks such as writing or summarizing primarily affects self-efficacy and cognitive reliance, whereas reliance on diagnostic or decision support systems may influence professional judgment and ethical reasoning. Consequently, the emotional and behavioral correlates of AI dependence, such as anxiety, stress, and depression, likely vary across application types. Future studies should differentiate between AI functions to better understand how domain-specific reliance shapes mental health and learning outcomes among students. Finally, future studies should test the proposed bidirectional model using longitudinal and mixed methods approaches, examining how personal traits and academic or digital contexts influence these reciprocal effects. Viewing generative AI dependence as a dynamic psychological process rather than a one-way phenomenon offers a more nuanced framework for understanding its mental health implications.

### Conclusions

This study conceptualized and empirically evaluated generative AI dependence as a specific form of digital behavioral reliance and examined its association with anxiety and depression among medical students. In contrast to previous studies that have centered on AI acceptance, ethical attitudes, or general technology overuse, this study focused on measurable psychological dependence on generative AI and its mental health correlates within a high-demand academic environment. By generating original data from a Latin American university context, this study advances the emerging literature at the intersection of digital behavior, medical education, and mental health. From a practical perspective, these findings highlight the importance of integrating digital literacy training, ethical AI use guidelines, and student mental health programs to prevent maladaptive AI reliance and foster healthier, more autonomous learning practices in real-world educational settings.

## Abbreviations

AI: artificial intelligence
DASS-21: Depression, Anxiety, and Stress Scale–21
DIA: Dependence on Artificial Intelligence Scale
RR: rate ratio
STROBE: Strengthening the Reporting of Observational Studies in Epidemiology
